# Supplementary material for: Suppressed macrophage response to quorum-sensing-active Streptococcus pyogenes occurs at the level of the nucleus
Source: bioRxiv. 2025 Feb 8:2025.02.07.637189. Preprint. [Version 1] doi: 10.1101/2025.02.07.637189 (PMC11839041; doi:10.1101/2025.02.07.637189)
Supplement: Supplement 1 [file NIHPP2025.02.07.637189v1-supplement-1.pdf]

bioRxiv preprint doi: https://doi.org/10.1101/2025.02.07.637189; this version posted February 8, 2025. The copyright holder for this preprint (which was not certified by peer review) is the author/funder, who has granted bioRxiv a license to display the preprint in perpetuity. It is made available under aCC-BY 4.0 International license.

Supplemental

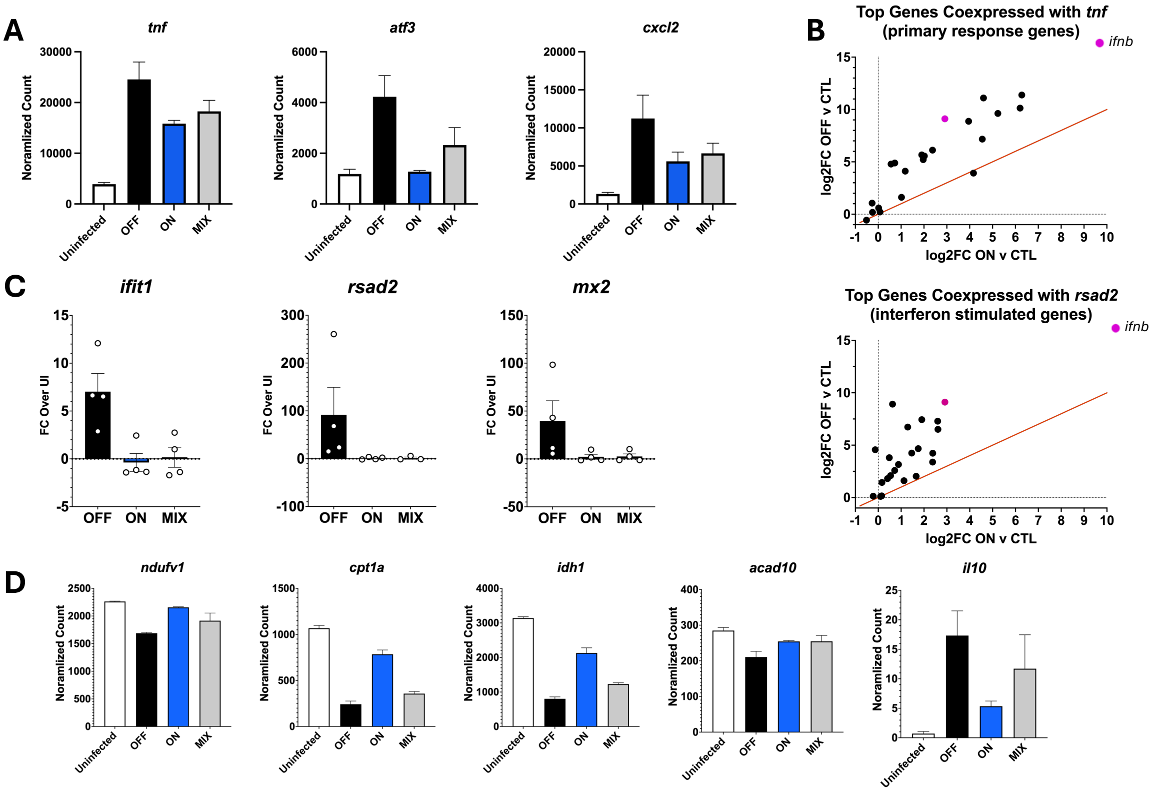

Figure S1.

A) Normalized read counts of infected RAW 264.7 cells at 2h post-infection (p.i.) of select early response genes from RNA-seq.

B) Comparison of log_2 fold changes of QS-OFF-infected over uninfected samples, and to QS-ON-infected over uninfected samples, averaged across all time points from RNA-seq dataset for genes most commonly co-expressed with *tnf* and *rsad2*. Genes most commonly co-expressed with these genes were determined using ARCHS4's RNA-seq gene-gene co-expression matrix acessed via Enrichr. Orange line shows the line of identity.

C) RT-qPCR of select type I interferon stimulated genes showing fold change over uninfected cells at 4h p.i.

D) Normalized read counts at 8h p.i. from RNA-seq dataset of select genes associated with oxidative phosphorylation and fatty acid metabolism, and *il10*.

bioRxiv preprint doi: https://doi.org/10.1101/2025.02.07.637189; this version posted February 8, 2025. The copyright holder for this preprint (which was not certified by peer review) is the author/funder, who has granted bioRxiv a license to display the preprint in perpetuity. It is made available under aCC-BY 4.0 International license.

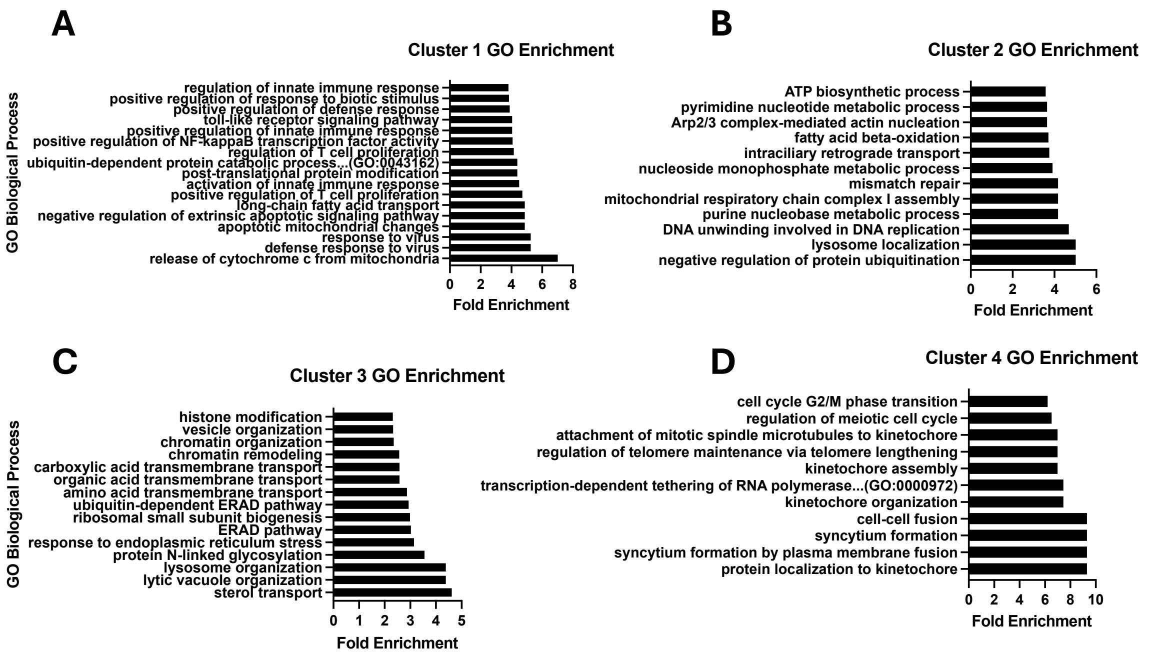

Figure S2. Enrichment of gene ontology (GO) biological processes clusters I-IV (A-D, respectively) created from analysis of RNA-seq dataset at 8h post-infection.

bioRxiv preprint doi: https://doi.org/10.1101/2025.02.07.637189; this version posted February 8, 2025. The copyright holder for this preprint (which was not certified by peer review) is the author/funder, who has granted bioRxiv a license to display the preprint in perpetuity. It is made available under aCC-BY 4.0 International license.

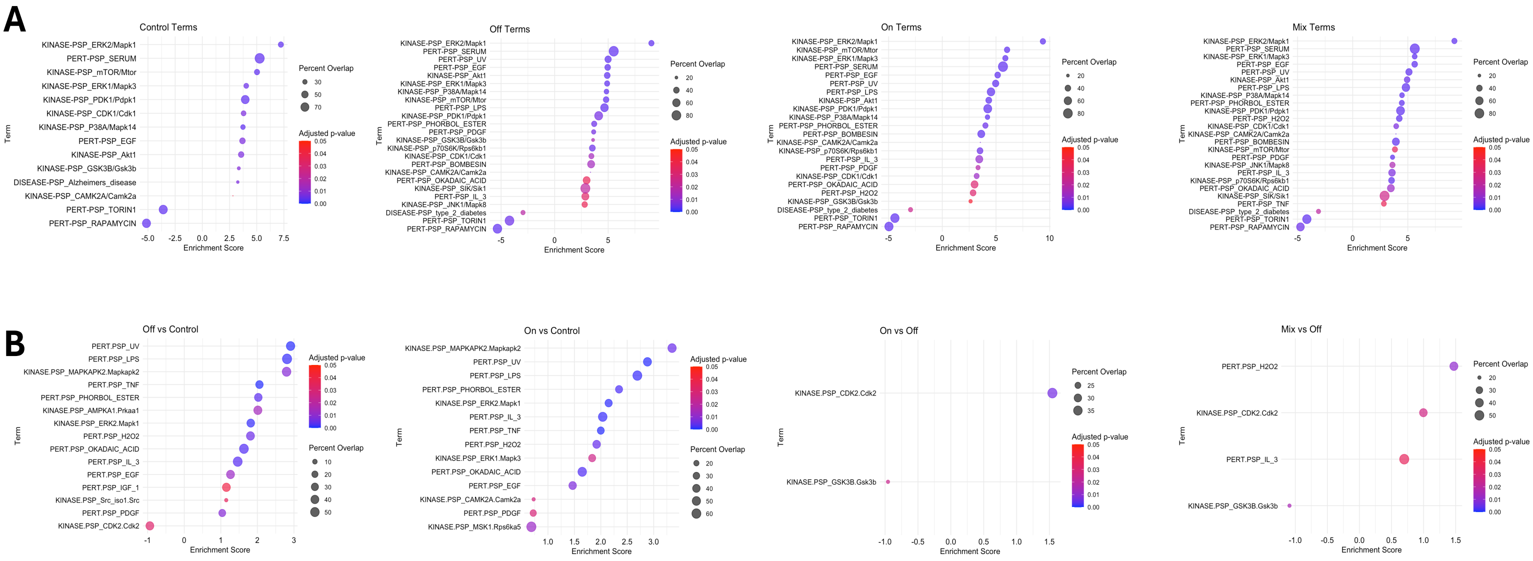

Figure S3.

A) PTM-SEA pathways that are significantly enriched (fdr-adjusted p-value<.05) from the post translational modification scan of each infection condition

B) PTM-SEA pathways that show significant differences in enrichments scores (p-value<0.05) in select pairwise comparisons. Significance determined by one way ANOVA.

bioRxiv preprint doi: https://doi.org/10.1101/2025.02.07.637189; this version posted February 8, 2025. The copyright holder for this preprint (which was not certified by peer review) is the author/funder, who has granted bioRxiv a license to display the preprint in perpetuity. It is made available under aCC-BY 4.0 International license.

Supplemental Table 1. Primers used for RT-qPCR.

**Primer**	**Sequence**
*gnl1*_F (housekeeping)	GCGGAAGCGAGGGCT
*gnl1*_R (housekeeping)	CCTGGGAAGGTTGCTGGTT
*tnf*_F	TGGAACTGGCAGAAGAGGCAC
*tnf*_R	TAGAGGCTGAGACATAGGCACCG
*rsad2*_F	CCCCGTGAGTGTCAACTACC
*rsad2*_R	TCTTCTCCAAACCAGCCTGT
*ifit1*_F	CCAAGTGTTCCAATGCTCCT
*ifit1*_R	GGATGGAATTGCCTGCTAGA
*mx2*_F	GTGGCAGAGGGAGAATGTCG
*mx2*_R	CTCGTCCACGGTACTGCTTT
*nos2*_F	GTTCTCAGCCCAACAATACAAGA
*nos2*_R	GTGGACGGGTCGATGTCAC
*cxcl2*_F	TCCAAAAGATACTGAACAAAGGCA
*cxcl2*_R	GCACATCAGGTACGATCCAG
*arg1*_F	ATTGGCTTGCGAGACGTAGA
*arg1*_R	GGCCTTTTCTTCCTTCCCAG
*klf4*_F	GTGCCCCGACTAACCGTTG
*klf4*_R	GTCGTTGAACTCCTCGGTCT
